# A fast adaptive algorithm for computing whole-genome homology maps

**DOI:** 10.1093/bioinformatics/bty597

**Published:** 2018-09-08

**Authors:** Chirag Jain, Sergey Koren, Alexander Dilthey, Adam M Phillippy, Srinivas Aluru

**Affiliations:** 1 School of Computational Science and Engineering, Georgia Institute of Technology, Atlanta, GA, USA; 2 Genome Informatics Section, Computational and Statistical Genomics Branch, National Human Genome Research Institute, National Institutes of Health, Bethesda, MD, USA; 3 Institute of Medical Microbiology, University Hospital of Düsseldorf, Düsseldorf, Germany

## Abstract

**Motivation:**

Whole-genome alignment is an important problem in genomics for comparing different species, mapping draft assemblies to reference genomes and identifying repeats. However, for large plant and animal genomes, this task remains compute and memory intensive. In addition, current practical methods lack any guarantee on the characteristics of output alignments, thus making them hard to tune for different application requirements.

**Results:**

We introduce an approximate algorithm for computing local alignment boundaries between long DNA sequences. Given a minimum alignment length and an identity threshold, our algorithm computes the desired alignment boundaries and identity estimates using kmer-based statistics, and maintains sufficient probabilistic guarantees on the output sensitivity. Further, to prioritize higher scoring alignment intervals, we develop a plane-sweep based filtering technique which is theoretically optimal and practically efficient. Implementation of these ideas resulted in a fast and accurate assembly-to-genome and genome-to-genome mapper. As a result, we were able to map an error-corrected whole-genome NA12878 human assembly to the hg38 human reference genome in about 1 min total execution time and <4 GB memory using eight CPU threads, achieving significant improvement in memory-usage over competing methods. Recall accuracy of computed alignment boundaries was consistently found to be >97% on multiple datasets. Finally, we performed a sensitive self-alignment of the human genome to compute all duplications of length ≥1 Kbp and ≥90% identity. The reported output achieves good recall and covers twice the number of bases than the current UCSC browser’s segmental duplication annotation.

**Availability and implementation:**

https://github.com/marbl/MashMap

## 1 Introduction

Algorithms for inferring homology between DNA sequences have undergone continuous advances for >3 decades, mainly in the direction of achieving better accuracy to compare distant genomes, as well as better compute efficiency to scale with growing data. Up until the last decade, reconstruction of a complete reference genome through sequencing and assembly was deemed a major landmark in genomics (Lander *et al.*, 2001; Venter *et al.*, 2001). However, it did not take long for high-throughput sequencing technologies to fuel population-wide genomics projects through low-cost genome assemblies (e.g. the Genome 10 K project, Haussler *et al.*, 2009). Analysis of these new genome assemblies, for both population-scale biological studies and timely diagnosis in clinical settings, requires faster and memory-efficient algorithms for facilitating whole-genome comparisons.

It is well-known that computing local alignments using an exact dynamic programming algorithm at the whole-genome scale is computationally prohibitive. This bottleneck motivated the development of seed-and-extend based genome aligners. Within the seed-and-extend paradigm, the two common approaches adopted to compute exact matches are either implemented using a hash table for *k*-mers (e.g. Altschul *et al.*, 1997; Ma *et al.*, 2002; Schwartz *et al.*, 2003; Yorukoglu *et al.*, 2016) or suffix trees and its variants (Brudno *et al.*, 2003; Bray *et al.*, 2003; Delcher *et al.*, 1999; Marçais *et al.*, 2018; Vyverman *et al.*, 2013). A third category includes cross-correlation based algorithms (e.g. Satsuma by Grabherr *et al.*, 2010). However, these approaches still remain computationally intensive. For instance, Nucmer (Kurtz *et al.*, 2004) and LAST (Kiełbasa *et al.*, 2011), two widely used genome-to-genome aligners, require 10 or more CPU hours to align a human genome assembly to a human reference genome.

The primary motivation behind this work is to develop a new genome-to-genome mapping algorithm that is fast and memory-efficient while maintaining accuracy on par with sensitive aligners. We seek a new problem formulation that also provides a convenient handle for users to specify how diverged the input genomes are, based on their knowledge of which organisms are being compared, expected quality of genome assembly, and sensitivity requirements of any further downstream biological analysis.

The inspiration behind our algorithmic strategy stems from recent developments in techniques for long-read analyses. MinHash-based estimation of Jaccard similarity of *k*-mer sets between DNA sequences has been adopted for state-of-the-art long read genome assembly (Koren *et al.*, 2017) and long read mapping (Jain *et al.*, 2017). Through our previous work Mashmap (Jain *et al.*, 2017), we demonstrated that a MinHash-based approximate mapping algorithm can compute long-read mapping boundaries with accuracy on par with alignment-based methods, while exhibiting two orders of magnitude speedup. Mashmap operates by assuming an error-distribution model, links alignment identity to Jaccard similarity, and provides probabilistic guarantees on output sensitivity. However, this algorithm is limited to end-to-end mapping of input sequences, which makes it impractical for contig mapping or *split-read* mapping. Here, we introduce new algorithmic strategies to compute local alignment boundaries for both whole-genome and split-read mapping applications.

Given minimum identity and length requirements for local alignments, we formulate the characteristics of homologies we intend to compute. Our new algorithm internally makes use of our previous end-to-end approximate read mapping framework (Jain *et al.*, 2017) by applying it to non-overlapping substrings of the query sequence. We mathematically show that all valid local alignment boundaries, which satisfy the user-specified alignment identity and length thresholds, are reported with high probability. Further, we formulate a heuristic to prioritize mappings with higher scores. We leverage the classic plane-sweep technique from computational geometry to develop an O(nlog⁡n) algorithm to solve the filtering problem, with *n* being the count of total mappings.

We demonstrate the practical utility of our algorithm Mashmap2 by evaluating accuracy and computational performance using real data instances, which include mapping mammalian genome assemblies and ultra-long nanopore reads to the reference genomes, and sensitive self-alignment analysis of the human genome. We compared the performance of Mashmap2 against a recent fast alignment-free method Minimap2 (Li, 2018) and the widely used alignment-based method Nucmer (Kurtz *et al.*, 2004; Marçais *et al.*, 2018). Mashmap2 operates in about a minute and 4 GB memory, including both indexing and mapping stages, to map human genome assembly to a reference when given minimum alignment identity and length requirements of 95% and 10 Kbp, respectively. This makes it one of the most resource-efficient software for genome-to-genome mapping, especially with respect to the memory-usage. This performance is achieved while maintaining output sensitivity percentage in the high 90 s. We also demonstrate its direct applicability in computing all ≥1 Kbp long duplications in the human genome with high accuracy. We expect that the performance and sensitivity guarantees provided by our algorithm will allow fast evaluation of draft assemblies versus a reference genome, scalable construction of whole-genome homology maps, and rapid split-read mapping of long reads to large reference databases.

## 2 The Mashmap2 algorithm

We designed Mashmap2 to enable fast computation of homology maps between two sequences or a sequence and itself. It consists of two algorithmic components. The first computes approximate boundaries and alignment scores for all pairs of substrings that exceed a user specified length and identity threshold. The second applies a novel filtering algorithm to optionally weed out redundant, paralogous mappings.

### 2.1 Computing local alignment boundaries

Consider all local mappings of the form Q[i..j] between sequences *Q* (query) and *R* (reference) of length *l*_0_ or more, such that Q[i..j] aligns with a substring of *R* with per-base error-rate $\leq {\text{ε}}_{max}$ and $|j-i+1|\geq l_{0}$. Alignment algorithms have quadratic time complexity, therefore an exact evaluation of the local mappings between all possible substring combinations will require at least Ω(|Q||R|) time. As such, solving this problem exactly is computationally prohibitive for typical sizes of real datasets. Instead of explicitly computing all such structures, we seek at least one inexact seed mapping of length $l_{0}/2$ along the path of each optimal alignment. Doing so, while maintaining high sensitivity and sufficient specificity will allow computation of the local alignments efficiently using an appropriate alignment algorithm.

In our approach, we leverage our previous alignment-free end-to-end read mapping algorithm, designed for mapping noisy long reads (Jain *et al.*, 2017). This allows us to benefit from its attractive properties including probabilistic guarantees on quality, and algorithmic and space efficiency. We continue to assume the same error model that was used in this work, also restated here. We assume that alignment errors, i.e, substitutions and indels in a valid alignment, occur independently and follow a Poisson distribution. We also simplify by assuming that *k*-mers are independent entities in sequences. For a given per-base error rate threshold ${\text{ε}}_{max}$, the read-mapping algorithm reports all target mapping coordinates and identity estimates of a read in the reference, where it aligns end-to-end with $\leq {\text{ε}}_{max}$ per-base error rate, with high probability. This is achieved by linking Jaccard coefficient between the *k*-mer spectra of the read and its mapping region to the alignment-error rate, under the assumed error distribution model.

#### 2.1.1 Proposed algorithm

**Fig. 1.**
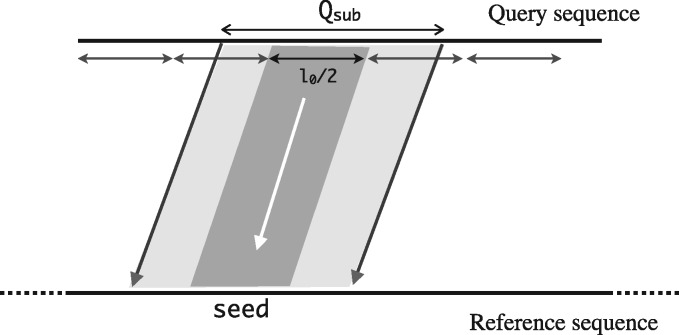
A local alignment depicting the inclusion of a length $l_{0}/2$ fragment of the query sequence

We first split the query sequence *Q* into $l_{0}/2$ sized non-overlapping fragments. If a substring of *Q*, say *Q_sub_*, of length $\geq l_{0}$ aligns against a substring of *R* with $\text{ε}\leq {\text{ε}}_{max}$ per-base error rate, then following statements hold true:

- There is at least one $l_{0}/2$ sized query fragment that maps end-to-end along the optimal alignment path. This is because at least $\left\lfloor \left(|Q_{sub}|-l_{0}/2+1\right)/(l_{0}/2)\right\rfloor \geq 1$ fragments completely span *Q_sub_* (see Fig. 1).
- Under the assumed error distribution, the expected count of errors in a sub-interval is proportional to its length. Therefore, the above $l_{0}/2$ sized fragment should map along the optimal alignment path with $\text{ε}\cdot l_{0}/2$ expected errors.

**Fig. 2.**
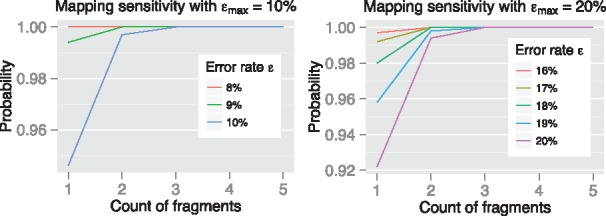
Probability of mapping at least one seed fragment for two different error-rate thresholds ${\text{ε}}_{max}=10\%,20\%$. As true error rate ε decreases, the probability values accordingly improve as expected. Similarly, longer alignments spanning more fragments are more likely to be reported. Most importantly, all the sensitivity scores are consistently above 90%. To compute the probability values, sketch size for Minhash based Jaccard estimation was assumed as 200, and the *k*-mer size was set to 16. These parameter values are internally computed by Mashmap (Jain *et al.*, 2017)

Accordingly, the read mapping routine in Mashmap can be used to map each fragment with ${\text{ε}}_{max}$ error-rate threshold. Let *p* be the probability that a fragment is mapped to the desired target position on the reference, computed as described by Jain *et al.* (2017). Probability of reporting at least one seed mapping along the optimal alignment is given by $1-{\left(1-p\right)}^{\left\lfloor \left(|Q_{sub}|-l_{0}/2\;+1\right)/(l_{0}/2)\right\rfloor }$. We show that these probability scores are sufficiently high, between 0.92 and 1.00 for alignment error rate thresholds ${\text{ε}}_{max}$ of 10% and 20%, respectively (Fig. 2).

The above seed matches and their alignment identity estimates are further processed to compute approximate local boundaries and their scores. After computing all seed matches, matches which involve consecutive query sequence fragments are merged together if they are mapped closely in the same order on the reference sequence. Suppose mappings from the consecutive query fragments $q_{i},q_{i+1},\ldots ,q_{j}$ are mapped to reference positions with begin positions $p_{0},p_{1},\ldots ,p_{j-i}$, respectively, then they are grouped together as a local alignment segment if $p_{0}\leq p_{1}\leq \ldots \leq p_{j-i}$, and $p_{k+1}-pk\leq l_{0},\;[0\leq k<j-i]$. The alignment boundaries are estimated as the first and last mapping offsets of the group. The corresponding alignment scores are estimated as their average identity estimate multiplied by the sum of the fragment lengths. We use these alignment boundaries and the scores as input to a subsequent filtering algorithm.

### 2.2 A geometric algorithm for filtering alignments

Large mammalian genomes and plant genomes have abundant repetitive sequences. As a consequence, a large fraction of inferior mappings are reported due to paralogous genomic segments or false positive mappings resulting from simple sequence repeats. Furthermore, from a biological perspective, closely examining all alternative mappings may not be feasible. Therefore, different strategies are adopted to identify biologically relevant outputs. We formulate a filtering heuristic for our mapping application, and develop an O(nlog⁡n) algorithm to solve it. We also prove that Ω(nlog⁡n) runtime is necessary to solve this problem. The effectiveness of this algorithm on real genomic data is demonstrated later, in the Results section.

#### 2.2.1 Problem formulation

**Fig. 3.**
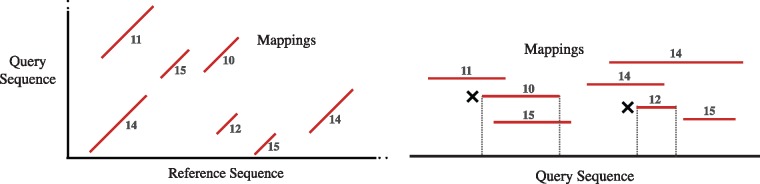
Left figure is a toy example to illustrate line segments corresponding to multiple local alignments obtained between a query and reference sequence. Each alignment segment is labeled with an alignment score. Suppose we want to filter best mappings for the query sequence. These segments are laid out as weighted intervals over the query sequence (right figure). In the above case, two intervals marked with a cross are completely subsumed by higher scoring intervals, and therefore, will be labeled as redundant by our filtering heuristic

Suppose all output mappings of a query sequence are laid out as weighted segment intervals, with the alignment scores used as weights (Fig. 3). We propose the following filtering heuristic: a segment is termed redundant if and only if it is subsumed by higher scoring segments at all of its positions. Therefore, the objective is to identify all *good* (non-redundant) segments. In practice, there can be multiple alignments with equal scores. Therefore, segment scores are allowed to be non-unique.

A sub-optimal $O(n^{2})$ algorithm for solving the above problem can be readily developed by doing an all to all comparison among the segments. However, it would lead to practically slow implementation for typical input sizes. The formulated filtering problem bears resemblance to the line segment intersection test problem for which Shamos and Hoey (1976) gave a classic O(nlog⁡n) algorithm using plane-sweep technique. Accordingly, we summarize their algorithm next, and subsequently describe the modifications made to solve the filtering problem.

#### 2.2.2 The Shamos–Hoey algorithm

Similar to the filtering problem, the problem of detecting whether *n* segments have an intersecting pair has a trivial $O(n^{2})$ solution. Shamos and Hoey solved this problem using a plane-sweep based O(nlog⁡n) time algorithm. The algorithm defines an ordering between segments in the 2D plane. The main loop of the algorithm conceptually *sweeps* a vertical line from left to right, and while doing so, the sweep-line status data-structure ℒ dynamically holds segments which intersect the sweep-line. The sweep-line halts at 2*n* endpoints of the input segments, and the order of segments in ℒ is evaluated to detect any intersection. For efficiency, this algorithm chooses a balanced tree to implement the sweep-line status ℒ. As such, it spends O(log⁡n) time at each halting point, and therefore, the total runtime is bounded by O(nlog⁡n). This algorithm is popular not only for its theoretical and practical efficiency, but also for ease of implementation.

In our problem as well, evaluating segments which intersect the vertical sweep-line at 2*n* endpoints is sufficient to identify all *good* segments. However, evaluating all intersecting segments at each endpoint is inefficient, and again leads to a quadratic algorithm. Therefore, we devise a new ordering scheme among segments which will enable us to evaluate only a subset of intersecting segments at each endpoint.

#### 2.2.3 Proposed algorithm for alignment filtering

We define an order between segments as follows: Between two segments, the segment with higher score is considered as greater, but if the scores are equal, then the segment with the latter starting position is considered as greater. This particular ordering helps avoid redundant computations, and will be crucial for bounding the runtime later.

Similar to the Shamos–Hoey algorithm, we also use a height-balanced Binary Search Tree (BST) as the data-structure for the sweep-line status ℒ, which tracks the segments that intersect the vertical sweep line. ℒ is required to support the following operations in our algorithm:

1. *insert*(*s*). Insert segment *s* into ℒ.
2. *delete*(*s*). Delete segment *s* from ℒ.
3. *mark_good*(). Mark all segments with highest score as *good* in ℒ.

Note that the *insert* and *delete* operations are naturally supported in O(log⁡n) time in BSTs, whereas the *mark_good* function can be realized as a sequence of *maximum* and *predecessor* operations. If there are *k* segments with equal and highest scores in ℒ, the function *mark_good* uses O(klog⁡n) time. With the data-structures and the operations defined above, we give an outline of the complete filtering procedure in Algorithm 1. The main loop of the algorithm iterates over the 2*n* segment endpoints, which is analogous to the sweep line moving from left to right, halting at the 2*n* points. In each iteration, we update the sweep-line status ℒ so that it holds the segments which intersect the sweep line, and mark the highest-scoring segments as *good* using the *mark_good* function.

**Lemma 1.**

Algorithm 1 solves the filtering problem correctly.

**Algorithm 1. Plane-sweep based alignment filtering algorithm**

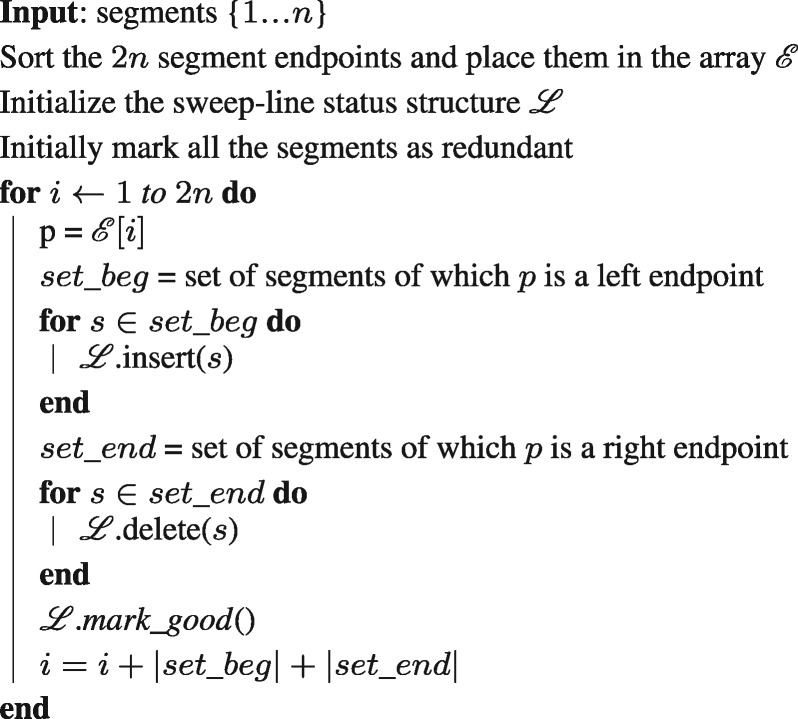

**Proof.**

Consider a function $S:N\to {\left\{0,1\right\}}^{n}$ from positions in the query sequence to subsets of segments {1,2,…,n}. A segment $s_{i}\in \{S(pos)\}$ if and only if it is among the highest scoring segments which overlap with the query sequence at position *pos.* Clearly, a union of all subsets in the domain of function *S* equals the set of *good* segments. If we perform a linear scan on the domain, from begin to end position of the query sequence, then value of *S* can change only at the 2*n* endpoints of the segments. Therefore, the highest scoring segments overlapping at the 2*n* endpoints constitute the set of *good* segments, which is precisely what Algorithm 1 computes.□

We make an additional modification to the above algorithm for efficiency, specifically in the *mark_good* function. In this function, we mark the highest scoring segments in the tree ℒ as *good.* We execute this by traversing the segments in decreasing order in ℒ, starting from the maximum. However, we terminate the traversal if a segment is observed as marked *good* already. This helps to avoid redundant computations, and the algorithm still remains correct due to the following property:

**Lemma 2.**

Consider all the segments with equal and highest scores in ℒ: $s_{1},s_{2},\ldots ,s_{j},\ldots ,s_{k}$, ordered in non-increasing manner. Suppose segment s_j_ has been marked good in one of the previous iterations of the algorithm, then the segments $s_{j+1},s_{j+2},\ldots s_{k}$ must have already been marked good as well.

**Proof.**

The aforementioned property is satisfied by default during the first iteration of the algorithm because there cannot be any previously marked segments. Suppose this property remains true till iteration *i*, and we are currently executing iteration *i* + 1. Segments $s_{1},s_{2},\ldots s_{k}\in ℒ$, so we know that the sweep line intersects these segments. Also, the ordering of the segments is maintained based on their scores and begin positions, and since the scores of these segments are equal, therefore $begin\_pos(s_{1})\geq$$begin\_pos(s_{2})\geq \ldots$$\geq begin\_pos(s_{k})$. Now consider the iteration when segment *s_j_* was marked *good.* Then, the sweep line must have intersected the segments $s_{j+1},s_{j+2},\ldots s_{k}$ as well. Therefore, if the segment *s_j_* was marked, then the segments $s_{j+1},s_{j+2},\ldots s_{k}$ must have been marked within or before the same iteration.□

The total cost of sorting, *insert* and *delete* operations in Algorithm 1 is clearly O(nlog⁡n). Because the revised *mark_good* function marks at most *n* segments throughout the algorithm, its runtime is also bounded by O(nlog⁡n). Thus, we conclude that the runtime complexity of our alignment filtering algorithm is bounded by O(nlog⁡n).

**Theorem 1.**

Given n alignment segments, Algorithm 1 solves the alignment filtering problem in O(nlog⁡n) time.

**Theorem 2.**

The above proposed filtering algorithm is optimal given the objective function.

**Proof.**

The INTEGER ELEMENT UNIQUENESS problem (given *n* integers, decide whether they are all unique) is known to have a lower bound of Ω(nlog⁡n) assuming the algebraic decision-tree model (Lubiw and Rácz, 1991). A simple transformation can be designed to show that

$$\text{INTEGER ELEMENT UNIQUENESS }\propto_{n}\text{ ALIGNMENT FILTERING }$$

Let $\left\{x_{1},x_{2},\ldots ,x_{n}\right\}$ be a set of *n* integer elements. For each element *x_i_*, construct a segment with begin position, end position and score as *x_i_*, *x_i_* and *i,* respectively. Because each segment is assigned a unique score, all the *n* elements are unique if and only if the filtering algorithm reports all the segments as *good.*

### 2.3 Related work for filtering alignments

There can be many alternative formulations of the filtering criteria. For instance, BLAST (Altschul *et al.*, 1997) filters out alignments if they are fully contained in ≥K alignments of higher scores (Berman *et al.*, 1999). Berman *et al.* also discussed a weaker alternative filtering condition where a match is filtered out if each position in a segment is covered by ≥K segments of higher score. Note that our filtering formulation is its special case with *K* = 1. They discussed a different O(nlog⁡n) time algorithm to solve the problem based on interval-tree of all input segments. Although a direct performance comparison is not possible due to unavailability of their implementation, the tree size in our plane-sweep based algorithm is limited by the number of overlapping segments which intersect the vertical sweep-line, which can be (and typically is) orders of magnitude smaller than the total count for large datasets. As such, even with the same theoretical complexity, we expect our algorithm to perform faster with less memory usage in practice.

### 2.4 Execution for mapping applications

The above filtering criteria is useful to identify the promising alignments between query and reference genomes. For the genome-to-genome mapping application, we execute the filtering algorithm twice, once to filter best alignments for query sequence, followed by filtering best alignments for reference sequence. Mappings which pass both filters constitute the orthologous matches, required for building a one-to-one homology map. For read mapping however, filtering on just the query sequence is appropriate. Accordingly, Mashmap2 provides two filtering modes: one-to-one and map for the two applications, respectively.

## 3 Results

We assess the performance of Mashmap2 for genome-to-genome and split-read mapping in comparison to recent versions of state-of-the-art software Minimap2 (Li, 2018) and Nucmer (Marçais *et al.*, 2018). Results indicate that Mashmap2 provides output of comparable quality, and yields significant gains in memory-usage. Subsequently, we demonstrate the utility of Mashmap2 in accurately computing all 1 Kbp long duplications in the human genome.

### 3.1 Genome-to-genome mapping

#### 3.1.1 Datasets

To evaluate and compare Mashmap2 for mapping genomes, we used six datasets D1–D6 listed in Table 1. Dataset D1 includes comparison between microbial genomes *E. coli* O157: H7 and *E. coli* K12. The two instances D2 and D3 require mapping of NA12878 human reference genome assemblies to the hg38 human reference genome. Query genome assemblies in both instances D2 and D3 are the recently published assemblies computed using Canu (Koren *et al.*, 2017), using ultra-long Oxford Nanopore Technology (ONT) reads (Jain *et al.*, 2018). Dataset D3 includes a long-read only Canu assembly whereas assembly in dataset D2 is also error-corrected using Illumina reads. The next two datasets D4, D5 involve inter-species genome comparisons- human vs. gorilla and chimp vs. gorilla, respectively. Finally, to evaluate Mashmap2 for the split-read mapping task, D6 includes raw ultra-long human ONT reads, generated using a single flowcell (Jain *et al.*, 2018). We restrict our benchmarking to real data instances because simulations typically fail to capture the full complexity of mutational processes.

**Table 1.** List of datasets used for evaluation

Id	Query sequences (≥10 Kbp)			Reference genome
	Source	# Sequences	N50 (bp)	
D1	*E. coli* O157 genome	2	5.5 M	*E. coli* K12 MG1655
D2	Human genome assembly (ONT+Illumina)	2269	7.7 M	Human (hg38)
D3	Human genome assembly (ONT)	2263	7.4 M	Human (hg38)
D4	Human (hg38) genome	365	145 M	Gorilla (gorGor5)
D5	Chimp (panTro5) genome	3086	137 M	Gorilla (gorGor5)
D6	Ultra-long human ONT reads	7656	129 K	Human (hg38)

*Note*: Datasets D1–D5 are included to evaluate Mashmap2 for genome-to-genome mapping application, and D6 for long read mapping application. We discarded a small fraction of contigs and reads with length <10 Kbp.

#### 3.1.2 Defining baseline and methodology

We used MUMmer package (v4.0.0.beta2), which includes the Nucmer4 alignment program for comparing DNA sequences (Marçais *et al.*, 2018). Nucmer4 is sensitive enough to report alignments for both assembly and read mapping tasks, therefore we considered its output as truth while evaluating accuracy. In addition, UCSC genome browser (Kent *et al.*, 2002) hosts high-quality pairwise syntenic alignment sets between popular mammalian genomes. Therefore, for evaluating the inter-species genome comparisons (D4, D5), we could use these as our truth sets. These alignments were originally computed using BLASTZ (Schwartz *et al.*, 2003) with careful parameter tuning and are more reliable for this purpose. We also used Minimap2 (v2.7-r659) (Li, 2018) as a baseline for various performance metrics. Minimap2 executes chaining algorithm on fixed-length exact matches to compute alignment boundaries. To our knowledge, it is among the fastest tools available to map DNA sequences in an alignment-free fashion.

Each software, including ours, exposes many parameters (e.g. *k*-mer or seed length). Default *k*-mer size in Mashmap2 is 16. We mostly conform to default parameters with all software tested, except as noted below. Mashmap2 mainly requires a minimum length and identity for the desired local alignments. In this test, we targeted long alignments, and accordingly fixed the minimum alignment length requirement as 10 Kbp. We set the minimum alignment identity requirement for all the datasets based on their input characteristics as {D1–D2: 95%, D3–D5: 90%, D6: 80%}. Accordingly, we tested Mashmap2 for reporting the alignment boundaries as per the provided requirements. Filtering modes were set to one-to-one and map for datasets D1–D5 and D6, respectively. Nucmer4 was run with default parameters, followed by running *delta-filter*, both components of the MUMmer package. Following its user documentation, *delta-filter* was executed with −1 parameter to construct one-to-one alignment map in datasets D1–D5 and –q parameter for read mapping in D6. Finally, Minimap2 supports genome-to-genome mapping mode using –x asm5 flag, and nanopore read mapping mode using -x map-ont. We executed all three software in multi-threaded mode using eight CPU threads. All comparisons were done on an Intel Xeon E5-2680 platform with 28 physical cores and 256 GB RAM.

#### 3.1.3 Runtime and memory usage

The wall-clock runtime and memory-usage of Mashmap2, Minimap2 and Nucmer4 using datasets D1–D6 are shown in Table 2. The runtimes represent end-to-end time, from reading input sequences to generating the final output. Minimap2 can report base-to-base alignments but does not do so by default. Thus, the final output of Mashmap2 and Minimap2 are alignment boundaries and scores, whereas Nucmer4 outputs base-to-base alignments. Both alignment-free methods Mashmap2 and Minimap2 are able to map most of the query bases to unique positions in all datasets (shown later), therefore base-to-base alignments can be computed quickly for the final output using chaining heuristics and vectorization techniques (Suzuki and Kasahara, 2018; Li, 2018).

**Table 2.** Total execution time and memory usage comparison of Mashmap2 against Minimap2 and alignment-based tool Nucmer4

Id	Mashmap2		Minimap2		Nucmer4	
	Time	Memory	Time	Memory	Time	Memory
D1	0.5 s	16 M	0.4 s	85 M	5.2 s	138 M
D2	1 m 26 s	3.5 G	3 m 3 s	17.3 G	5 h 1 m	53 G
D3	6 m 33 s	3.6 G	3 m 11 s	15.9 G	2 h 10 m	53 G
D4	27 m 33 s	9.0 G	15 m 6 s	26.7 G	33 h 4 m	57 G
D5	25 m 40 s	7.7 G	5 m 54 s	25.7 G	24 h 58 m	56 G
D6	13 m 6 s	10.0 G	3 m 10 s	10.4 G	25 m 2 s	53 G

*Note*: All software were run in parallel using eight CPU threads.

From Table 2, we observe that Mashmap2 uses significantly less memory when compared to Minimap2, while Minimap2 generally achieves better runtime. Mashmap2 improves memory-usage by 5.3x, 4.9x, 4.4x, 3.0x, 3.3x and 1.04x for the six datasets, respectively. The performance gap against Nucmer4 is much wider with speedups of 10.4×, 210×, 19.8×, 72.0×, 58.4× and 1.9×, and memory-usage improvements by 8.6×, 15.1×, 14.7×, 6.3×, 7.3× and 5.3× on the datasets D1–D6, respectively. Low memory requirements in Mashmap2 can allow for larger comparisons (e.g. a genome against a big, in-memory reference database).

**Fig. 4.**
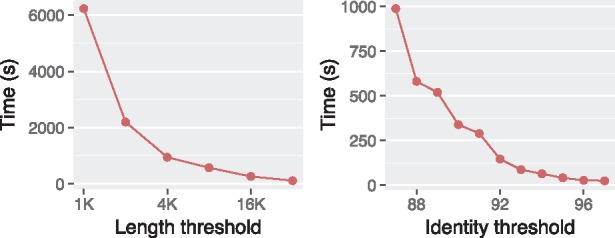
Wall time of Mashmap2 decreases with increasing length or identity thresholds using dataset D3 and eight CPU threads. In this experiment, identity and length thresholds were fixed to 90% and 10 Kbp while varying the other parameter. Memory-usage also follows a similar trend (data not shown)

Mashmap2 and Minimap2 follow the same initial step of sampling *k*-mers using minimizers (Roberts *et al.*, 2004; Schleimer *et al.*, 2003), followed by computing their exact matches in the reference genome. Mashmap2 is designed to identify all matches that meet the criteria, while Minimap2 is designed to find the best. This partly explains the differences observed in their running times. The optional filter in Mashmap2, if disabled, enables it to return all hits, e.g. for the use-case of finding all repeats (presented later in Section 3.2). Mashmap2 includes an efficient MinHash-based mechanism to estimate Jaccard similarity and auto-tunes its internal parameters (e.g. *k*-mer sampling rate, Jaccard similarity threshold), conforming to the local alignment identity and length requirements provided by the user. Auto-tuning can help achieve faster runtime and reduce memory-usage with increasing identity and length thresholds (Fig. 4). It is important to maintain high accuracy while being fast, therefore we next evaluate the quality of output.

#### 3.1.4 Accuracy

Accuracy evaluation of Mashmap2 and Minimap2 in comparison to the assumed truth sets is shown in Table 3. As stated before in Section 3.1.2, recall was measured against the assumed true alignments, i.e. Nucmer4 alignments for intra-species comparisons (D1–D3, D6) and UCSC browser pairwise alignments for inter-species comparisons (D4, D5) which satisfy the alignment requirements in terms of minimum length and identity provided to Mashmap2. We also expected Minimap2 to report these alignments because it is designed to compute matches in these identity ranges.

**Table 3.** Accuracy evaluation of Mashmap2 and Minimap2 to do an alignment-free computation of mapping boundaries

Id	Recall scores				Fraction of query bases mapped uniquely	Precision^a^
	Mashmap2 (%)	Minimap2 (%)	#True alignments	Mashmap2 (%)	Minimap2 (%)	Mashmap2 (%)
D1	100	100%	144	74.0	78.9	72.0
D2	97.5	98.3	35 186	96.8	96.3	50.0
D3	97.1	98.1	37 807	96.9	96.0	55.2
D4	97.0	97.7	63 908	87.5	91.3	75.9
D5	97.5	98.0	65 289	89.8	93.2	57.3
D6	99.3	99.5	4349	89.9	84.6	34.8

*Note*: Recall was measured against the truth sets assumed (Section 3.1.2).

^a^Fraction of mappings which satisfied alignment thresholds in Mashmap2.

A reported local alignment boundary estimate by Mashmap2 or Minimap2 was assumed to recall a true alignment if it overlapped with the alignment on both query and reference sequences, and if the mapping strand matched. From Table 3, we observe that both Mashmap2 and Minimap2 consistently achieved high recall scores ≥97%, with Minimap2 performing slightly better. Obtaining high recall scores by itself is not sufficient, because it can be achieved by mapping a query sequence to all possible positions. In parallel to achieving high recall scores, both Mashmap2 and Minimap2 mapped a large fraction of query genome assemblies to unique mapping positions in the reference genomes. To show this, we computed the fraction of base-pairs of the query sequence that are mapped to a single position on the reference genome (Table 3).

Next, we evaluated the precision, i.e. what fraction of Mashmap2 mappings yield one or more alignments above the specified length and identity thresholds. We used LAST (Kiełbasa *et al.*, 2011) to compute the alignments. The precision score of Mashmap2 averages to 57.5% across all the datasets, varying from 34.8% (in D6) to 75.9% (in D4). The corresponding scores for Minimap2 using the same threshold values are much lower (average = 15%), but Minimap2 follows different design principles and lacks similar guarantees on the characteristics of its output. In the current context of tasks that require such guarantees, Mashmap2 provides better precision on all datasets.

#### 3.1.5 Efficacy of the filtering algorithm

Eukaryotic genomes contain many repetitive sequences, therefore, the motivation behind our plane-sweep based filtering heuristic is to discard noisy mappings, and compute promising matches between the query and reference genomes. We show the importance and effectiveness of our filtering strategy in Table 4. Note that a large fraction of the initial mappings was pruned out by the filter. While doing so, high recall scores against the assumed true sets were maintained (see Table 4). Although we do not present the contribution of this phase to the total runtime, the plane-sweep algorithm is fast in practice; it used an insignificant fraction of the total runtime.

**Table 4.** Effectiveness of the filtering algorithm in Mashmap2

Id	Count of output mappings			Recall scores	
	Without filter	With filter	Ratio (without/with)	Without filter (%)	With filter (%)
D1	145	82	1.77	100.0	100.0
D2	6, 541, 930	3985	1642	99.9	97.5
D3	53, 331, 538	3137	17 001	99.7	97.1
D4	152, 536, 106	4756	32 072	100.0	97.0
D5	152, 266, 777	13 834	11 007	100.0	97.5
D6	18, 604, 261	12 930	1439	99.9	99.3

*Note*: A large fraction of mappings were filtered out by the algorithm, while the recall scores remained largely unaffected. Last column in this table is copied from Table 3 for convenience.

### 3.2 Computing duplications in the human genome

Soon after the publication of the human genome, it was realized that the genome is replete with repetitive sequences (International Human Genome Sequencing Consortium, 2004). Intra- and inter-chromosomal duplications have been found to play a vital role in genome evolution, its stability and diseases (Emanuel and Shaikh, 2001; Pu *et al.*, 2018), and knowing the location of such repeats can be important for many genomic analyses. Yet, fully annotating all repeats in a genome can be computationally challenging. To demonstrate the scalability of Mashmap2, we computed all ≥1 Kbp duplications in the human genome (GRCh38, Schneider *et al.*, 2017) with ≥90% alignment identity. The importance of these duplications has been known for a long time (Bailey *et al.*, 2002; Emanuel and Shaikh, 2001); accordingly the UCSC genome browser also maintains them as a public database (named as segmental duplications) for the human genome. The goal of our experiment is to recover as many duplications as possible. Due to the probabilistic guarantees provided by our algorithm (Section 2.1), we expect it to compute such duplications with a high recall value. Typical genome-to-genome aligners including Minimap2, Nucmer4 and BLASTZ do not provide such guarantees, and typically require extensive parameter tuning as well as preprocessing of input to perform this task (e.g. Bailey *et al.*, 2001, 2002). We show that Mashmap2 serves as a straight-forward and accurate solution to address such applications.

#### 3.2.1 Methodology

**Fig. 5.**
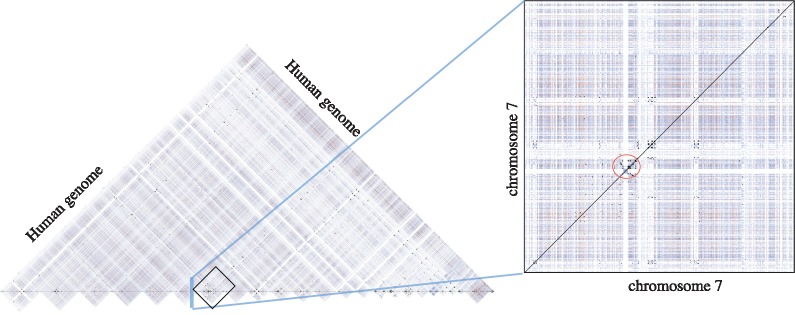
Visualization of ≥1 Kbp duplications in the human genome computed using Mashmap2. Alignments are colored based on their lengths: blue 1–5 Kbp, red 5–10 Kbp, black >10 Kbp. Majority of blue and red mappings occur due to SINEs and LINEs repeats, respectively. Right plot is a magnification of ≥1 Kbp duplications within chromosome 7. Chromosome 7 is known to be one of the most duplicated human chromosomes. Large clustered duplications in red circle are associated with Williams-Beuren syndrome (Hillier *et al.*, 2003)

We used 24 chromosome sequences (1–22, X, Y) and mitochondrial DNA from the hg38 version of the human genome as our input sequence set. To compute all ≥1 Kbp, ≥90% identity duplications, we directly used Mashmap2 with the same length and identity requirements, with filtering disabled. From its output, we discarded short (≤500bp) mappings with <90% estimated identity, plus the trivial duplications (i.e. regions matching with themselves), and were left with 2.1 billion candidate mappings. The count of reported mappings is high due to several high-copy repeat families in the genome, not all of which exceed our minimum thresholds. To remove the shorter or lower identity mappings, each of the approximate alignments was processed using LAST to compute a base-level alignment. This resulted in 210 million validated alignments with ≥1 Kbp length and ≥90% identity. We note that a large fraction of the candidate mappings failed to satisfy the specified cutoffs here. This is because Mashmap2 looks at the Jaccard similarity of *k*-mer sets to evaluate the mappings, but does not consider the distribution of *k*-mer match positions. As a result, frequently occurring exact repeats of length <1 Kbp in the human genome can also qualify as a match in the output. For example, an exact 300 bp *Alu* repeat induces many shared *k*-mers in a 1 Kbp window, resulting in an artificially high identity estimate for the larger window. It may be possible to improve the specificity by further considering the distribution of *k*-mer matches. This experiment took 120 CPU hours for executing Mashmap2 and 24 000 CPU hours for validating all reported mappings using LAST. We show a dot-plot visualization of the reported alignments in Figure 5, which appears dense due to extensive duplications in the human genome. Finally, we converted the alignments into BED format to compare against the UCSC database using Bedtools (Quinlan and Hall, 2010); the accuracy results are discussed next.

#### 3.2.2 Accuracy evaluation and insights

The UCSC Segmental Duplications database for the hg38 human genome was computed using a standard pipeline proposed by Bailey *et al.* (2001), and was last updated in 2014. It is important to note that prior to computing genomic duplications, their method removed high-copy repeat elements (e.g. LINEs, *Alu*s) from the genome. Therefore, this database is not an exhaustive set of all ≥1 Kbp, ≥90% identity duplications in the genome, but a significant fraction of them. Nonetheless, low-copy repeat annotations have a higher likelihood of being missed by a mapper. Therefore, checking the recall against this database serves as an appropriate test to evaluate Mashmap2 in computing all homologous mappings of the specified characteristics.

**Fig. 6.**
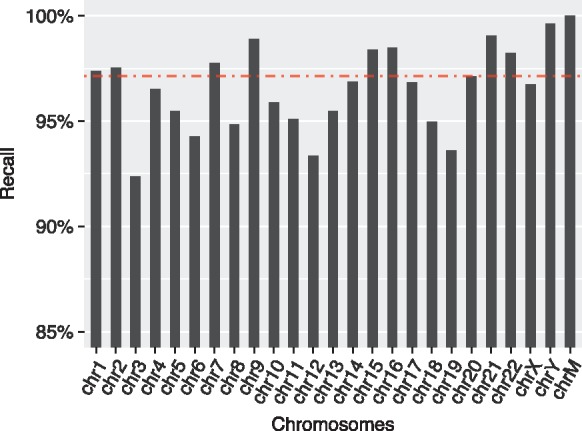
Recall scores of duplications computed using Mashmap2 against the UCSC segmental duplication database. Above 90% recall scores are achieved on each chromosome consistently. The red dotted line shows the aggregate recall score of 97.15% for the complete genome

To measure recall on each chromosome, we computed coverage of those UCSC duplication annotations that have overlap with Mashmap2 duplications, and divided it by the coverage of all UCSC duplication annotations. Therefore, a 100% recall score would imply that all base-pairs which are annotated as segmental duplication in the UCSC database are part of one or more Mashmap2 alignments. We show these recall scores for each chromosome as well for the complete genome in Figure 6. Recall is consistently observed to be above 90% for each chromosome, and the aggregate recall for the complete genome is 97.15%. Among the 2.85% missed alignments, a large fraction of alignments were not recalled because difference in the alignment parameters can affect alignment identity and length. As a result, same regions can yield slightly different alignments using LAST and BLAST. If we relax the alignment identity and length cutoff in LAST to 88% and 950 bp, respectively, the recall score improves to 98.28%. High recall scores achieved here, as well as in our prior experiments, demonstrate high sensitivity of our algorithm for any specified alignment characteristics by the user, which is consistent with the theory in Section 2.1.

**Fig. 7.**
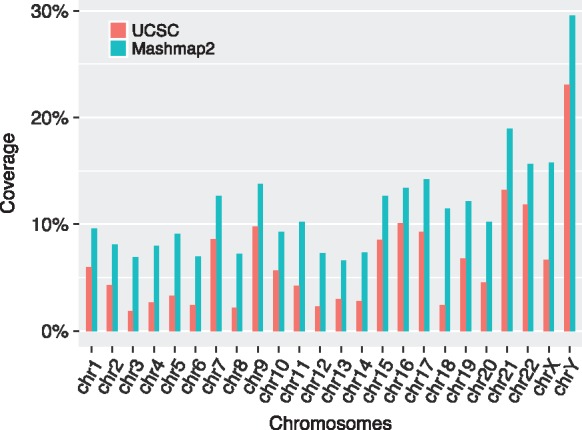
Comparison of genomic coverage between the UCSC Segmental Duplication database and Mashmap2 output alignments. Both methods reported equal coverage 83% on mitochondrial chromosome (not shown above to keep the plot legible). Coverage of duplications computed using our method is significantly higher, owing to its exhaustive search of all repeats with ≥1 Kbp length and ≥90% identity without repeat masking

Finally, we compared the coverage of our alignments versus the UCSC database. Since our method did an exhaustive search of all duplications with ≥1 Kbp length and ≥90% identity without masking any genomic repeats, we observe that our algorithm attains either equal or higher coverage on each chromosome (Fig. 7). For the complete genome, coverage of our alignments is 10.3%; 5% higher than the coverage of UCSC annotations. We further examined the subset of our duplications which do not overlap with UCSC segmental duplications. Indeed a large coverage fraction (82%) comprises of high-copy repeats (i.e. coverage depth >50), potentially due to common repeat elements, which explains the wide gap in the coverage observed. The remaining 18% coverage fraction, however, is composed of low-copy repeats, with coverage depth ≤50 indicating the potential to uncover novel segmental duplications. Validating this possibility requires a more careful inspection of the output, and will be our future work. Mashmap2 alignments are available online at https://gembox.cbcb.umd.edu/mashmap/index.html.

## 4 Discussion

In this work, we presented a fast algorithm for computing homology maps between whole genomes. We have given both theoretical and experimental evidence of the sensitivity provided, in terms of computing local alignment boundaries based on the minimum alignment length and identity parameters. To the best of our knowledge, this is the first practical and scalable algorithm to provide such guarantees. This formulation grants a convenient mechanism for users to execute this algorithm based on the underlying applications, including (but not limited to) mapping genome assemblies of variable quality, aligning long reads to reference genomes, or computing segmental duplications in large genomes. Additionally, we formulated a filtering heuristic, and proposed an optimal plane-sweep based filtering algorithm for prioritizing alignments based on their scores and locations. The filtering algorithm is practically fast, accurate and easy to implement in few lines of code using standard libraries. When mapping a human genome assembly to the human reference genome, Mashmap2 takes only about a minute from reading input sequences to generating the final alignment boundaries, identity estimates, and a dot-plot for visualization. Because of the underlying auto-tuning mechanism in Mashmap2, performance depends on the sensitivity requirements provided to the algorithm. As the pace of whole-genome sequencing continues to increase, faster practical algorithms and theoretical advances will help analyze available and forthcoming data.

Although our algorithm optimizes mapping of a single genome assembly to a single reference genome, its runtime would scale linearly when mapping to multiple reference genomes. Planned future work includes development of sub-linear algorithms using existing ideas of non-linear reference genome representations. We also plan to evaluate biological novelty of the human segmental duplications computed in this work.
